# Supplementary material for: 1,4-α-Glucosidase from Fusarium solani for Controllable Biosynthesis of Silver Nanoparticles and Their Multifunctional Applications
Source: Int J Mol Sci. 2023 Mar 20;24(6):5865. doi: 10.3390/ijms24065865 (PMC10057468; doi:10.3390/ijms24065865)
Supplement: Supplementary file 1 [file ijms-24-05865-s001.zip › Supplementary material.pdf]

Supporting Information

1,4-α-glucosidase from Fusarium solani for controllable bio-synthesis of silver nanoparticles and their multifunctional ap-plications

Ying-Jie Zeng[1], Xiao-Ling Wu[2], Hui-Rong Yang[1], Min-Hua Zong[2], Wen-Yong Lou[*2]

[1] College of Food Science & Technology, Southwest Minzu University, Chengdu, 610041, Sichuan, China

[2] Laboratory of Applied Biocatalysis, School of Food Science and Engineering, South China University of Technology, Guangzhou 510640, Guangdong, China

*Correspondence should be addressed to wylou@scut.edu.cn

[*] Correspondence should be addressed to wylou@scut.edu.cn

Synthesis of AgNPs using the protein obtained from ammonium sulfate precipitation method

The protein fractions precipitated with saturated ammonium sulfate solution in a final saturation of 50%, 60%, 70% and 80% could be not applied to synthesise AgNPs. However, when the coenzyme DPNH and *β*-NADPH were added to reaction system, AgNPs could be formed (Figure S1a). Due to some black precipitate existing in the AgNPs solution with DPNH addition, thus the *β*-NADPH was the suitable coenzyme that was benefit for AgNPs synthesis. Moreover, the protein fraction precipitated with saturated ammonium sulfate solution in a final saturation of 80% exhibited the excellent AgNPs effect. Therefore, the 80% protein fraction was further purified by ion exchange chromatography (IEC) (Figure S1c).

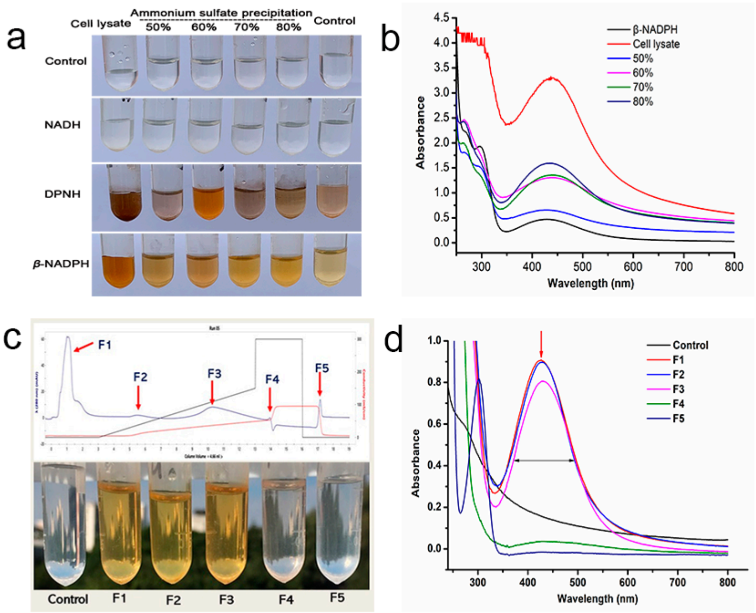

Figure S1. AgNPs synthesized by protein fractions precipitated with saturated ammonium sulfate solution in a final saturation of 50%, 60%, 70% and 80% with or without coenzyme addition (a); UV–Vis spectra of AgNPs synthesized by protein fractions with or without *β*-NADPH addition (b); 80% protein fraction further purified by ion exchange chromatography (IEC) and AgNPs synthesized with different collected purified fractions (c); UV–Vis spectra of AgNPs synthesized by purified protein fractions with or without *β*-NADPH addition (d).

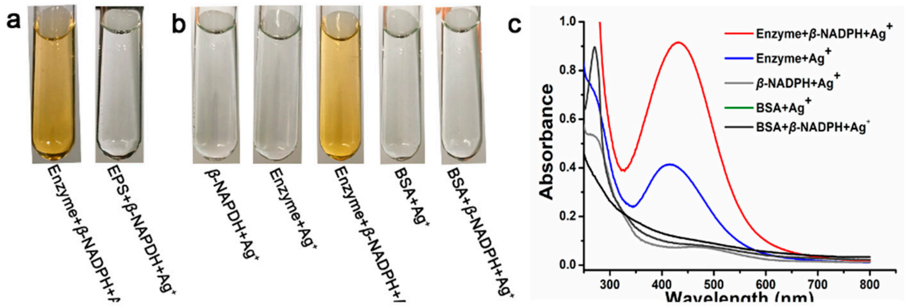

Figure S2. (a) Color change of AgNPs synthesized in purified protein or exopolysaccharides (EPS) solutions in presence of β-NADPH. (b) Comparative color change of AgNPs synthesized in purified protein and BSA solutions in presence/absence of β-NADPH. (c) UV–Vis spectra of AgNPs synthesized by BSA, EPS, and purified protein.

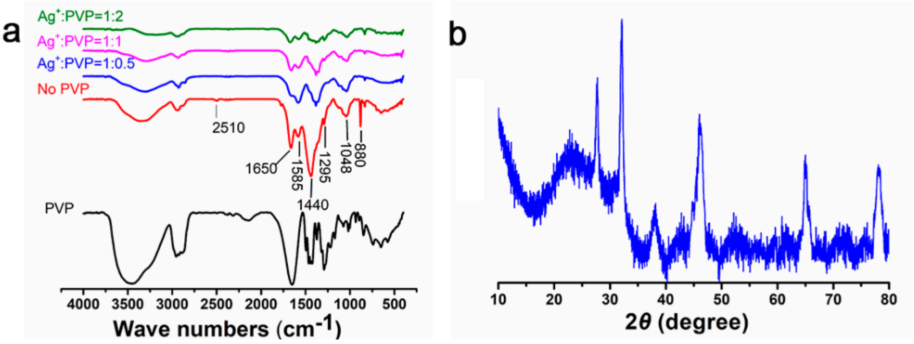

Figure S3. FTIR spectra of PVP and AgNPs synthesized with different mass ratio of Ag^{+}/PVP (a); X-ray diffraction (XRD) of AgNPs with Ag^{+}/PVP mass ratio of 1:1 (b).

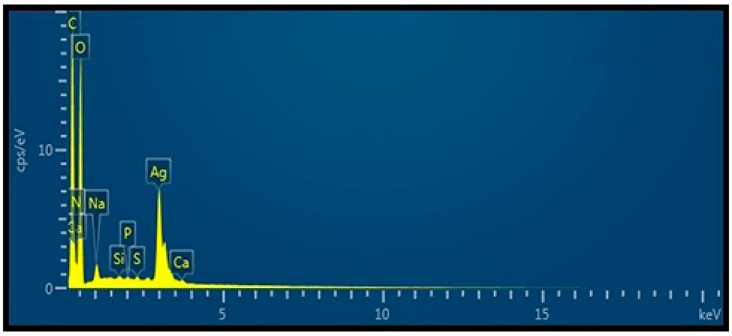

Figure S4. Energy dispersive spectrometer (EDS) of AgNPs synthesized in presence of PVP.

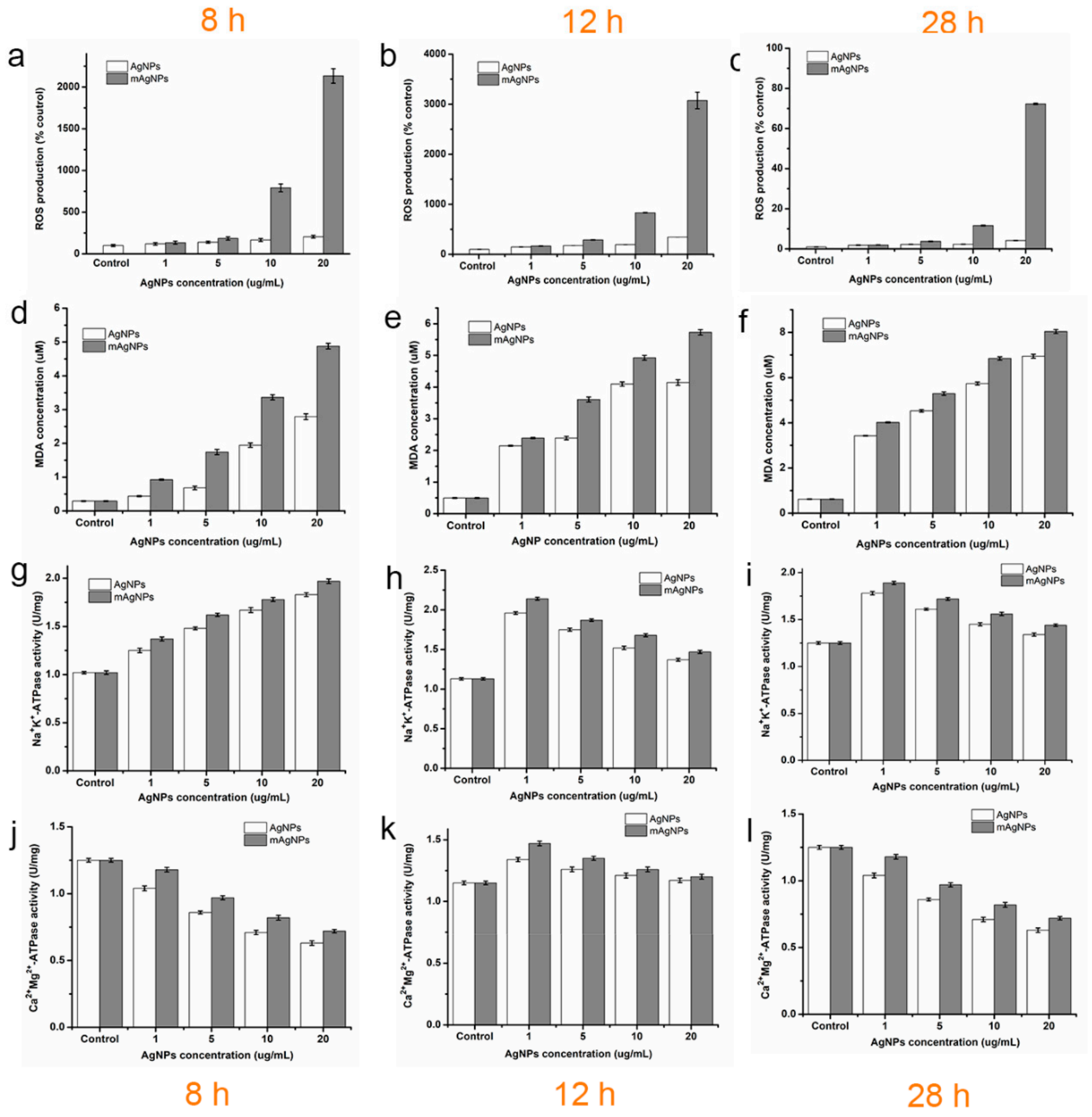

Figure S5. Effects of different concentrations (0, 1, 5, 10 and 20 ug/mL) of AgNPs and mAgNPs (AgNPs synthesized with the Ag$^+$/PVP mass ratio of 1:1) on ROS contents of *E. coli* cells at 6 h, 12 h and 28 h. ROS levels of *E. coli* at 6 h (a), 12 h (b), and 28 h (c); MDA concentrations of *E. coli* at 6 h (d), 12 h (e), and 28 h (f); Na$^+$K$^+$-ATPase activity of *E. coli* at 6 h (g), 12 h (h), and 28 h (i); Ca^{2+}Mg^{2+}-ATPase activity of *E. coli* at 6 h (j), 12 h (k), and 28 h (l).

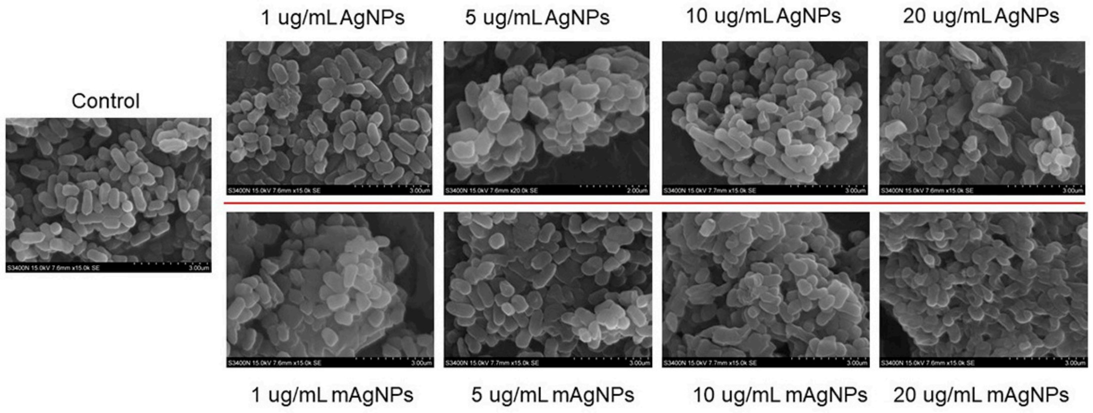

Figure S6. SEM images of *E. coli* treated with different concentrations AgNPs or mAgNPs.

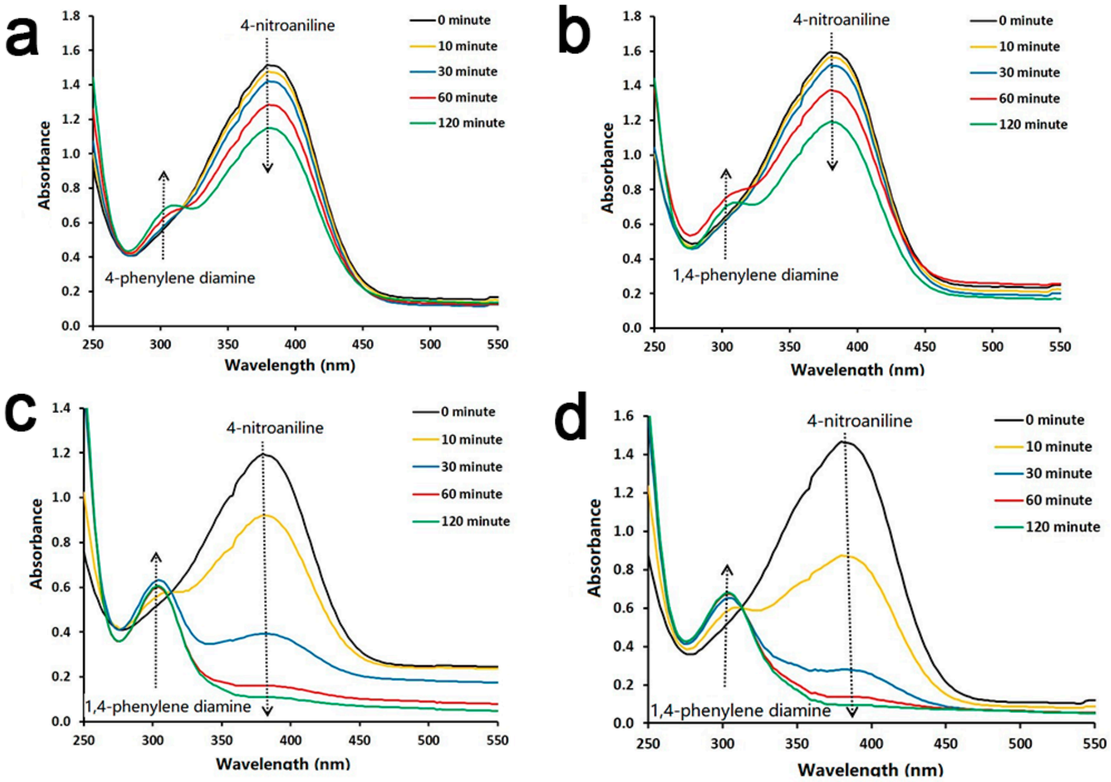

Figure S7. Absorbance spectra for p-nitro-aniline after addition of the so-obtained Ag-Au nano-alloy as function of time: blank (a), 1,4-α-glucosidase(b), AgNPs (c) and AgNPsM (d).
